# An Epigenetic Compound Library Screen Identifies BET Inhibitors That Promote HSV-1 and -2 Replication by Bridging P-TEFb to Viral Gene Promoters through BRD4

**DOI:** 10.1371/journal.ppat.1005950

**Published:** 2016-10-20

**Authors:** Ke Ren, Wei Zhang, Xiaoqing Chen, Yingyu Ma, Yue Dai, Yimei Fan, Yayi Hou, Ren Xiang Tan, Erguang Li

**Affiliations:** 1 Medical School and State Key Laboratory of Pharmaceutical Biotechnology, Nanjing University, Nanjing, China; 2 Jiangsu Laboratory of Molecular Medicine, Medical School, Nanjing University, Nanjing, China; 3 The Hoffmann Institute of Immunology, Guangzhou Medical University, Guangzhou, China; 4 Nanjing University of Chinese Medicine, Nanjing, China; University of Wisconsin-Madison, UNITED STATES

## Abstract

The human HSV-1 and -2 are common pathogens of human diseases. Both host and viral factors are involved in HSV lytic infection, although detailed mechanisms remain elusive. By screening a chemical library of epigenetic regulation, we identified bromodomain-containing protein 4 (BRD4) as a critical player in HSV infection. We show that treatment with pan BD domain inhibitor enhanced both HSV infection. Using JQ1 as a probe, we found that JQ1, a defined BD1 inhibitor, acts through BRD4 protein since knockdown of BRD4 expression ablated JQ1 effect on HSV infection. BRD4 regulates HSV replication through complex formation involving CDK9 and RNAP II; whereas, JQ1 promotes HSV-1 infection by allocating the complex to HSV gene promoters. Therefore, suppression of BRD4 expression or inhibition of CDK9 activity impeded HSV infection. Our data support a model that JQ1 enhances HSV infection by switching BRD4 to transcription regulation of viral gene expression from chromatin targeting since transient expression of BRD4 BD1 or BD1/2 domain had similar effect to that by JQ1 treatment. In addition to the identification that BRD4 is a modulator for JQ1 action on HSV infection, this study demonstrates BRD4 has an essential role in HSV infection.

## Author Summary

The human HSV-1 is associated with cold sore, while HSV-2 is considered a pathogen of sexually transmitted infection. Lytic infection by HSV-1 and HSV-2 triggers cellular responses as the virus strives to express its own genes and to replicate. To investigate host factors involved in the lytic infection cycle, we screened a chemical library of epigenetic regulation and identified several BET bromodomain inhibitors that enhanced both HSV-1 and HSV-2 infection. Using JQ1, a well-defined BRD4 inhibitor, as a model we showed that JQ1 increases HSV infection by allocating BRD4 to viral gene promoters. We also showed that BRD4 regulates HSV-1 and HSV-2 lytic infection by recruitment of factors for transcription elongation. The study expands the knowledge on viral replication regulation and identifies novel targets for antiviral agents.

## Introduction

Herpes simplex virus-1 and -2 (HSV-1, HSV-2) are important pathogens of human diseases [1,2]. HSV-1 infection is mainly associated with cold sores and blisters, while HSV-2 is a major factor of sexually transmitted infections [3,4]. Patients acquire HSV-1 at relatively young ages, while initial HSV-2 infections occur mainly after puberty, often transmitted after intimate contact [5]. It has been estimated that two thirds of adult population aged 15–49 are infected with HSV-1, while over 550 million individuals aged 15–49 have genital infection with HSV-1 or HSV-2 [1,2].

HSV-1 and HSV-2 are double-stranded DNA viruses that are genetically similar and share many common features in infection and replication. The viruses are acquired initially by direct contact and replicate within mucosal epithelial cells. In the meantime, the virion can enter the nerve termini of sensory neurons and travel transgradely to the cell bodies and establish latency. Latent infection serves as a reservoir of virus for recurrent infection and transmission to other individuals. Although immeasurable advances have been made towards our understanding of HSV infection, the molecular machinery responsible for HSV replication regulation remains elusive and largely mystified.

Multiple viral and cellular factors are involved in HSV replication [6]. Upon HSV infection of epithelial cells, more than 80 viral genes are sequentially expressed in a temporal cascade, including the immediate early genes, early genes and late genes. Meanwhile, the HSV genome is rapidly incorporated into nucleosomes bearing histone modifications that resemble characteristics of heterochromatic structures [7,8]. Histone modifications have an essential role in HSV lytic and latent infections. For example, chemicals that inhibit histone deacetylase activity are reported to enhance viral replication [9,10]. Inhibition of the histone demethylase LSD1 blocks virus lytic replication and reactivation from latency [11,12]. Whether other factors of epigenetic regulation have a role in HSV infection is not well studied.

We took an approach by screening a chemical library of epigenetic regulation to identify factors affecting HSV infection. The library consists of well-defined inhibitors of HDAC, methyltransferase, the aurora kinase, among other categories. In addition to TSA, a known HDAC inhibitor that has been reported to enhance HSV-1 and HSV-2 infectivity, we discovered several structurally different BRD4 inhibitors that promoted HSV-1 and HSV-2 infection. BRD4 is a member of the bromodomain and extraterminal (BET) family, which includes BRD2, BRD3, BRD4 and BRDT in mammals. BRD4 is an epigenetic reader and recruits transcriptional regulatory complexes to acetylated chromatin and therefore participates in host gene regulation [13] and has multiple functions in HPV transcription activation and infection [14–17]. BRD4 interacts with HIV Tat protein to negatively regulate HIV-1 replication [18]. There has been no previous report on BRD4 participation in HSV infection. We therefore performed detailed studies and discovered that bromodomain inhibition enhances HSV infection by promoting transcription factor association with HSV gene promoters.

## Results

### A library screening identifies BRD4 inhibitors that promote HSV infection

**Fig 1:**
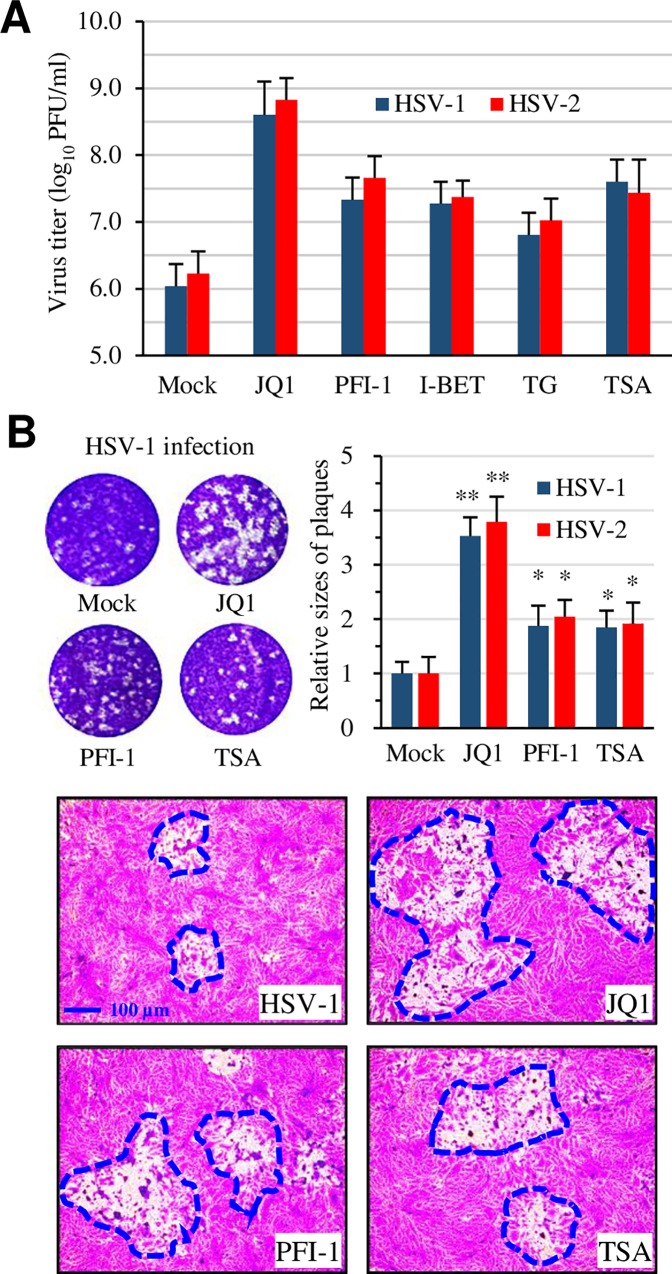
BET domain inhibition enhances HSV-1 and HSV-2 production. (A) Titration of infectious virus production by plaque assay. Vero cells in triplicate were treated with a test compound or with 0.1% DMSO (Mock) at 2 hr prior to inoculation. HSV-1 or HSV-2 at 1 pfu/cell (MOI = 1) was used. The compounds were left throughout the infection. The samples were harvested at 24 hr PI and used to titrate infectious virus by plaque assay. The concentrations are as the following: JQ1 at 300 nM, PFI-1 at 500 nM, I-BET-762 (I-BET) at 1 μM, TG101348 (TG) at 3 μM, and TSA at 150 nM. (B) Representative compounds JQ1 and PFI-1 on plaque sizes. Vero cells in 6-well plates were infected in the presence or absence of a test compound with approximately 50–100 pfu/well of HSV-1 or HSV-2. Wells from plaque assay were photographed and the sizes of 30 randomly selected plaques were measured using ImageJ. The relative sizes the plaques were plotted and presented as mean ± SD. Data were analyzed using paired T test for statistical significance. * denotes p<0.05, and ** as p<0.01.

To investigate whether an epigenetic factor(s) regulates HSV infection, we screened an epigenetic compound library. We first determined the toxic effect against Vero cells, a host cell line for the initial screening, even though the concentrations for those compounds to cause cytotoxic effect in tumor cells are well documented. The effect on HSV-2 infection was then tested by pre-treatment of Vero cells using the maximal non-toxic concentrations. In those studies, we also included trichostatin A (TSA) and acyclovir as controls since those compounds are known to either enhance or inhibit HSV-2 infection. If a compound showed an effect that resembled viral infection, we then performed secondary infection assays to quantitatively measure their effect on virus infection. We identified several candidates from a library of 129 compounds that promoted the cytolytic effect associated with HSV-2 infection, while no compound from this library showed inhibitory effect in the infection assay. In addition to HDAC inhibitors like TSA that are known to enhance HSV-2 infection, we also identified several BET bromodomain inhibitors, including JQ1, I-BET-762, PFI-1, and TG101348, with enhancement effect on HSV-2 infection. The effect was confirmed by the detection of increased infectious virus production using both HSV-1 and HSV-2 (Fig 1A). Concomitant with increases in viral production, plaque sizes in those samples were obviously increased, compared to mock-treated controls (Fig 1B). On average, the plaque sizes in JQ1-treated samples were increased by over 250%, while those in TSA-treated samples by approximately 55–60%. The names, PubChem CID, and their effect on HSV-1 and HSV-2 infection are listed in Table 1.

**Table 1:** Summary of names, PubChem IDs, and the minimal concentrations defined to enhance HSV-2 infection*. The epigenetics compound library contains 129 small molecule compounds that are classified into inhibitors of histone deacetylases (HDACi), lysine demethylases, histone acetyltransferases (HATs), DNA methyltransferases (Dnmts), and the epigenetic reader domain inhibitors, to name a few. Thirteen compounds showed enhancement effect on infection, while no compounds showed inhibitory effect. To quantitatively describe their effect on HSV-2 infection, we determined the concentration that would cause 20% changes in cell viability from HSV-2-infected controls. In this regard, the compounds were series diluted and tested on Vero cells in triplicated samples. An MOI of 1 was used for infections. HSV infection causes cytolytic effect that was determined at 48–60 hr PI by an MTT assay.

Name	PubChem CID	EC_20_ (nM)	Inhibitor of
Trichostatin A	444732	30	HDAC
Quisinostat	11538455	30	HDAC
CUDC-907	54575456	30	HDAC, PI3K
CUDC-101	24756910	100	HDAC
Givinostat	9804992	100	HDAC
HDAC-42	6918848	100	HDAC
Pracinostat	49855250	300	HDAC
Scriptaid	5186	300	HDAC
Tubastatin A	57336514	3000	HDAC
JQ1	46907787	50	BET bromodomain
PFI-1	71271629	100	BET bromodomain
I-BET-762	46943432	300	BET bromodomain
TG101348	16722836	1000	BET bromodomain, JAK

* The concentration that causes a 20% (EC_20_) reduction in OD readings was given as a minimal concentration required to enhance HSV infection.

### JQ1 enhances HSV-1 and HSV-2 infection dose-dependently

**Fig 2:**
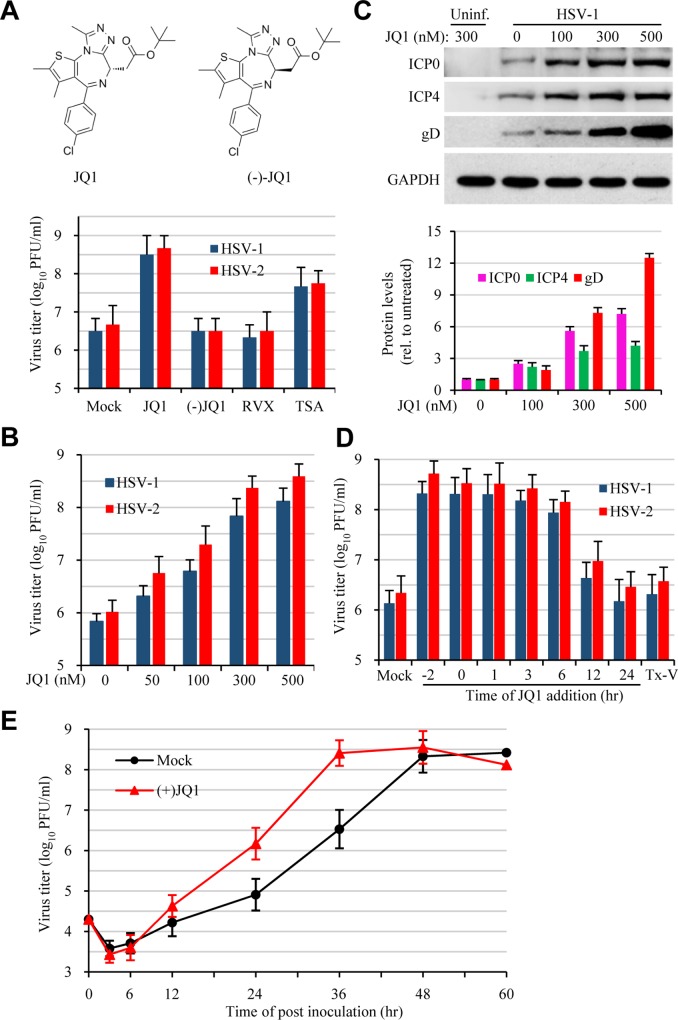
Bromodomain inhibition promotes HSV-1 and HSV-2 infection. (A) Effect of BD1 and BD2 inhibition on HSV-1 infection. Vero cells were treated with BD1 inhibitor JQ1 (300 nM), BD2 inhibitor RVX-208 (RVX, 500 nM), or HDAC inhibitor TSA (150 nM). (-)JQ1, an inactive enantiomer of JQ1, at 300 nM was also included as a control. The cells were infected with HSV-1 or HSV-2 (1 MOI) at 2 hr post treatment. The cells were harvested at 24 hr PI and used for plaque assay. The data are presented as mean ± SEM of triplicate samples. (B and C) Dose effect of JQ1 on HSV infection. Vero cells were treated with JQ1 at concentrations as indicated. HSV-1 or HSV-2 at 1 MOI were used. The samples were harvested at 24 hr PI for virus titration by plaque assay (B) or used for protein expression analysis by western blot (C). The bar graph represents quantitative measurement of viral protein expression (relative to infected but untreated controls). The experiment was performed independently 3 times. Data are presented as mean ± SEM of two independent measurements. (D) Time effect of JQ1 addition on HSV infection. HeLa cells were treated with JQ1 at 300 nM at time points as indicated. The time of inoculation was counted as 0, and -2 hr refers to 2 hr prior to infection. In parallel experiments, the virus was incubated with 300 nM JQ1 at RT for 1 hr and then used to infect the cells after dilution (Tx-V). The cells were infected with HSV-1 at 0.5 MOI for 36 hr and virus production was determined by plaque assay. (E) JQ1 accelerates HSV-1 infection. HeLa cells were mock-treated or treated with 300 nM JQ1 prior to HSV-1 infection (MOI = 0.3). The samples were harvested at times as indicated and virus production was titrated.

A literature review indicated that the abovementioned BET compounds are developed as anticancer and anti-inflammatory agents. They compete selectively for acetylated lysine residues against BET proteins, particularly BRD4, by occupying the acetyl-lysine binding pocket of the BD1, thus inhibit BET protein binding to acetylated histones and disrupt the formation of the chromatin complexes essential for host gene expression [19–22]. To delineate a mechanism of bromodomain inhibition on HSV infection, we focused on JQ1 since the target of this compound is well defined [19] and the inactive enantiomer (-)-JQ1 is also commercially available. To preliminarily determine whether BD domain inhibition was responsible for enhanced HSV infection, Vero cells were treated with JQ1, RVX-208, a BD2-specific inhibitor of BRD4 [23], or with the inactive (-)-JQ1, prior to HSV-1 or HSV-2 infection. Treatment with 300 nM JQ1, but not RVX-208 or (-)-JQ1, resulted in increased production of HSV-1 and HSV-2 (Fig 2A). The effect of JQ1 on HSV-1 and HSV-2 infection was dose dependent (Fig 2B), a conclusion validated by increases in viral protein expression (Fig 2C). Additionally, we found that the effect was not restricted to Vero cells since treatment of HeLa, HEp-2, SK-N-SH, or primary mouse embryonic fibroblast (MEF) cells with JQ1 or with PFI-1 also increased HSV-1 and HSV-2 production (Fig A in S1 Text). Since both Vero and HeLa cells responded to JQ1 equally well, we then used HeLa cells for subsequent studies.

Next, we determined the time effect of JQ1 addition on HSV-1 and HSV-2 infection using HeLa cells. We found that addition of JQ1 at 2 hr prior to (-2 hr), or within the first 6 hr of inoculation had similar effect on HSV infection. The effect became less significant when JQ1 was added at 12 hr and 24 hr PI (Fig 2D). In contrast, treatment of HSV (Tx-V) with JQ1 prior to inoculation had little effect on HSV infection, indicating the compound did not target the virion for its enhancement effect.

We also measured the course of virus production in the presence or absence of JQ1 (Fig 2E). During the course of the infection, HSV-2 titers in JQ1-treated samples were higher compared to those in vehicle-treated controls. Although the highest titers in JQ1 treated and untreated groups were at comparable levels, the highest titer in JQ1-treated samples was reached by approximately 12 hr than that in the controls. The titers started to drop, probably by cell lysis due to the infection. These results indicated that BD1 domain inhibition promoted infectious virus production.

### Knockdown of BRD4 expression limits HSV infection

**Fig 3:**
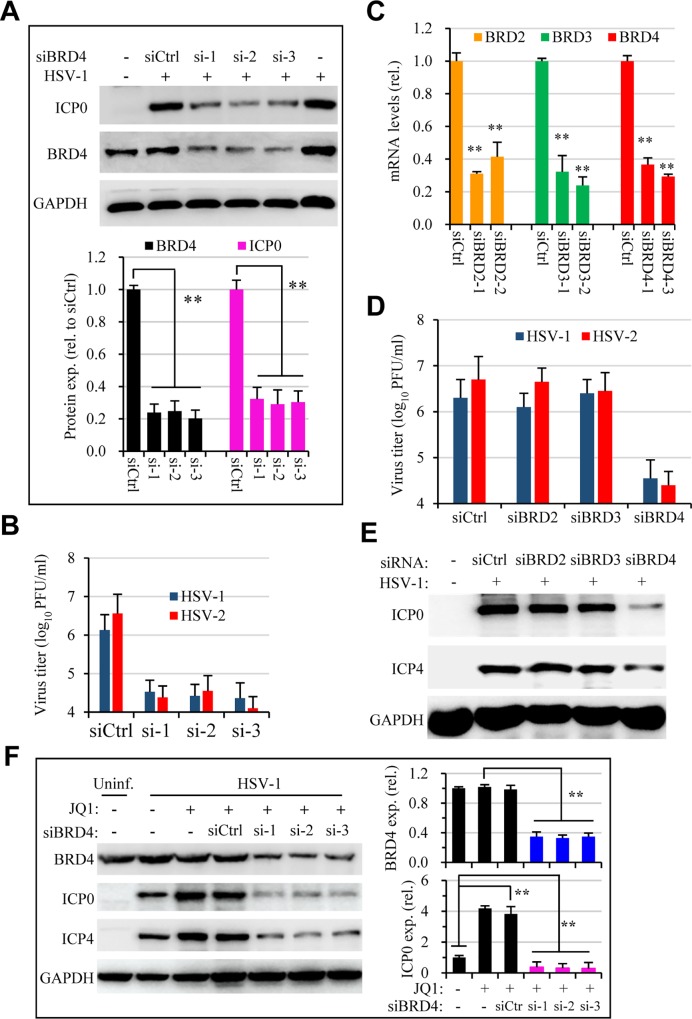
Dependency of BRD4 expression for HSV-1 infection and JQ1 activity. HeLa cells were transfected with a control siRNA (siCtrl) or with siRNA (si-1, -2, -3) targeting BRD4. The cells were then used for infection (HSV-1, 1 MOI) at 48 hr post transfection. Virus production was determined by plaque assay, while viral protein synthesis was determined at 24 hr PI by immunoblotting. (A) siRNA treatment on BRD4 expression and on HSV-1 infection determined by immunoblotting for viral ICP0 and cellular BRD4 expression. Bar graph representative measurement of BRD4 and ICP0 expression. ** denotes p≤0.01. The experiment was performed independently 3 times. (B) Knockdown of BRD4 expression on infectious virus production. Data are presented as mean ± SEM of duplicated samples. (C, D, E) Suppression of BRD4, but not BRD2 or BRD3, inhibits HSV infection. The effect of individual siRNAs on BRD2, BRD3, and BRD4 on corresponding gene expression in HeLa cells was validated by real-time PCR. Gene expression was normalized to GAPDH and expressed as relative levels to siCtrl (C). Suppression of BRD4, but not BRD2 or BRD3 inhibited virus infection, determined by titration study (D) and by immunoblotting for HSV-1 proteins (E). F. Suppression of BRD4 expression ablates JQ1 enhancement effect on HSV-1 infection. Bar graphs at the right represent quantitative measurement of BRD4 and ICP0 protein bands. JQ1 was tested at 300 nM.

Among the bromodomain proteins, JQ1 has been shown to target the BD domains of BRD4 with high affinity [19]. Despite this strong selectivity, we would like to verify that JQ1 promoted HSV infection through BRD4. In this regard, we performed RNAi studies to determine whether suppression of BRD4 expression would have similar effect on HSV infection. HeLa cells were treated with 3 different siRNAs targeting BRD4 expression or with a scrambled control (siCtrl). siRNA treatment significantly suppressed BRD4 expression (Fig 3A). When tested for HSV infection using those cells, we found unexpectedly that suppression of BRD4 expression inhibited virus production as was determined by titration and immunoblotting studies (Fig 3A and 3B). The effect was specific since knockdown of BRD4, but not BRD2 or BRD3, suppressed HSV-1 and HSV-2 infection (Fig 3C, 3D and 3E). Importantly, we found knockdown of BRD4 expression also ablated the enhancement effect of JQ1 on HSV infection (Fig 3F). It is known that c-Myc is among the downstream genes regulated by BRD4 and JQ1 treatment selectively down regulated c-Myc expression [24,25]. Indeed, c-Myc expression was down regulated in JQ1-treated and BRD4 siRNA treated samples (Fig B in S1 Text), suppression of c-Myc by siRNA did not impact HSV infection. Therefore, the results clearly showed that BRD4 expression had an essential role in HSV infection and JQ1 exerted its enhancement effect on HSV-1 and HSV-2 infection through BRD4. It is therefore interesting to delineate a mechanism governing BD1 inhibition on HSV infection.

### BRD4 truncation mutants on HSV infection

**Fig 4:**
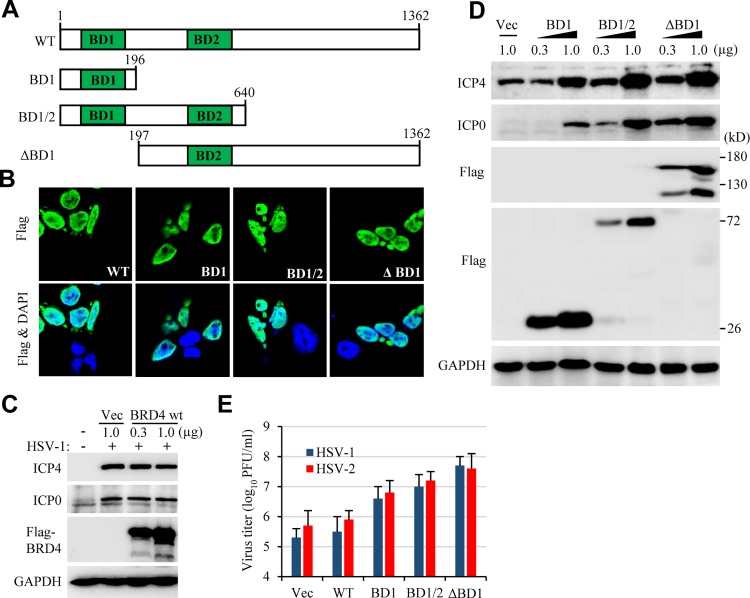
Overexpression of BRD4 mutants on HSV-1 infection. (A) Schematic drawing of BRD4 constructs. The DNA was subcloned into p3xFLAG-CMV-24 vector for mammalian cell expression. (B) BRD4 wt and its mutants were detected in the nuclei of transfected 293T cells. (C) Transient transfection of BRD4 wt had no effect on HSV-1 infection, determined by ICP0 and ICP4 expression. 293T cells in 6-well plates were transfected with p3xFLAG empty vector or with p3xFLAG-BRD4 wt at 0.3 and 1.0 μg for 24 hr. The cells were then infected with HSV-1 at 0.5 MOI for 24 hr. (D) Transient transfection of BD1, BD1/2 or ΔBD1 increases HSV-1 ICP0 and ICP4 expression. 293T cells were transfected and used as in (C). (E) BRD4 mutants promote HSV-1 and HSV-2 infection as were determined by plaque forming assay.

To firmly establish a role of BRD4 in HSV infection, we tested whether overexpression of BRD4 would lead to increases in HSV infection. To this end, the readily transfectable 293T cells were transfected with an empty vector or with a plasmid for BRD4 expression. Transient expression of full length BRD4 (BRD4 wt) did not increase HSV-1 production, even though the protein was expressed at high levels and localized correctly to the nuclei (Fig 4A, 4B and 4C). Since most cells we tested had high levels of endogenous BRD4 protein expression, we suspected that endogenous BRD4 was at sufficient levels in supporting HSV infection.

BRD4 is a reader protein and interacts with acetylated lysine residues using its BD domains. JQ1 binds to the BD domains and interrupts BRD4 interaction with acetyl lysine residues [19]. We reasoned that the BD domains might function similarly to JQ1 on HSV infection since BD domains may compete with BRD4 for acetyl lysine interactions. We therefore generated vectors for BD1, BD1/2 expression (Fig 4A). We also prepared BRD4 with BD1 deletion (ΔBD1) to potentially eliminate BRD4 chromatin targeting ability, and tested whether those mutants would support HSV infection. The truncated protein were mainly detected in the nuclei (Fig 4B) since they retain the putative nucleus localization signals. Similar to JQ1 treatment, BD1 or BD1/2 expression resulted in increased HSV infection as was demonstrated by immunoblotting and titration studies (Fig 4D and 4E). Impressively, BRD4 with BD1 deletion (ΔBD1) showed similar effect to JQ1 on HSV infection, suggesting the BD1-modulated events compete against BRD4 function on HSV infection. The results suggested to us that JQ1 promoted HSV infection by potentially diverting BRD4 function due to BD1 blocking.

### BRD4 recruits transcription regulatory factors and localizes to HSV promoters

**Fig 5:**
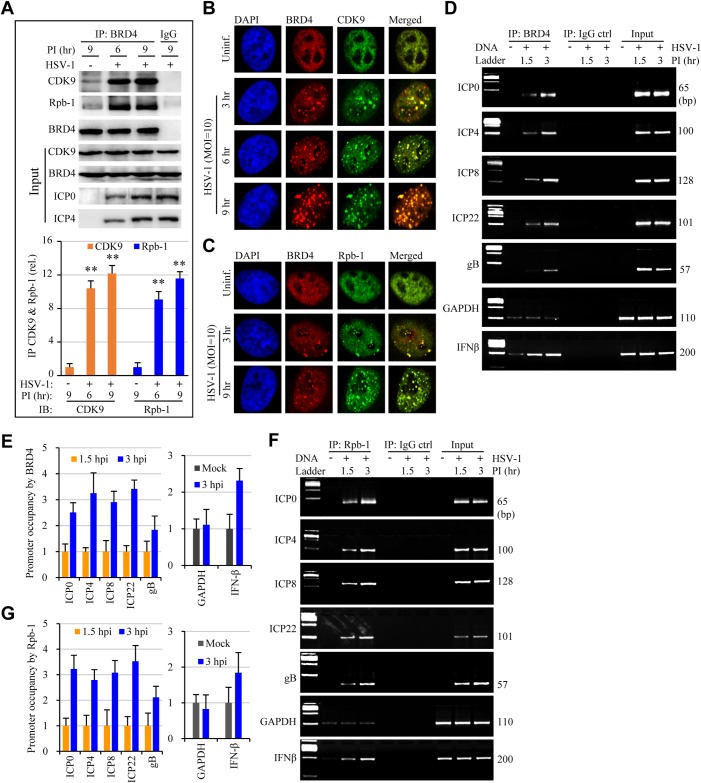
HSV-1 infection induces protein complex formation and the association with viral gene promoters. HeLa cells were infected with HSV-1 at 10 MOI for times as indicated. The samples were then processed for detection of protein association by immunoprecipitation assay, for protein co-localization by immunostaining, for protein-DNA interactions by a modified ChIP assay, respectively. (A) HSV-1 infection promotes protein association involving BRD4, CDK9, and RNAP II. Proteins in the lysates were used as control for input. The bar graph represents quantitative measurement of protein band intensities in anti-BRD4 immunocomplexes. The data are presented as mean ± SEM of 2 independent measurement. (B and C) Co-localization of BRD4 with CDK9 (B) and Rpb-1/RNAP II (C). The nuclei were stained using DAPI (blue). (D to G) Viral gene promoters in the BRD4 or Rbp-1/RNAP II immunocomplexes were determined by PCR (D, F) and real-time PCR (E, G). The GAPDH and IFNβ gene promoters in the complexes were included as controls for validation of ChIP assays.

In addition to participation in epigenetics regulation, BRD4 also regulates the transcription of cellular genes by recruiting of the positive transcription elongation factor P-TEFb [13,26,27] and transcriptional activators and repressors [28]. Next, we addressed whether JQ1 promoted HSV infection by allocating BRD4 complex more selectively to viral gene expression. We first studied whether HSV infection induced complex formation involving BRD4, P-TEFb, and RNAP II by immunoblotting for CDK9 and Rpb-1, subunits of P-TEFb and RNAP II, respectively. As shown in Fig 5A, a weak association between BRD4 with CDK9 and Rpb-1 was detected in the uninfected cells. HSV infection markedly induced BRD4 association with the two proteins (Fig 5A), an observation that was substantiated with immunostaining studies for protein co-localization (Fig 5B and 5C), indicating HSV infection promoted complex formation involving BRD4 and CDK9 or Rpb-1 proteins.

We then performed a modified ChIP assay to determine whether the complex was recruited to viral gene promoters. We used HSV-1 strain F for those studies since the genome sequence is readily accessible. There was selective association between BRD4 with viral gene promoters of IE, early and late genes that was demonstrated by PCR (Fig 5D) and quantitatively measured by qPCR (Fig 5E). The pattern of their association with BRD4 paralleled that with RNAP II (Fig 5F and 5G). The association was selective and specific, since primer pairs that anneal further upstream or within the coding region failed to detect viral DNA from the anti-BRD4 immunocomplexes (Fig C in S1 Text).

### JQ1 enhances transcriptional factor association with viral gene promoters

**Fig 6:**
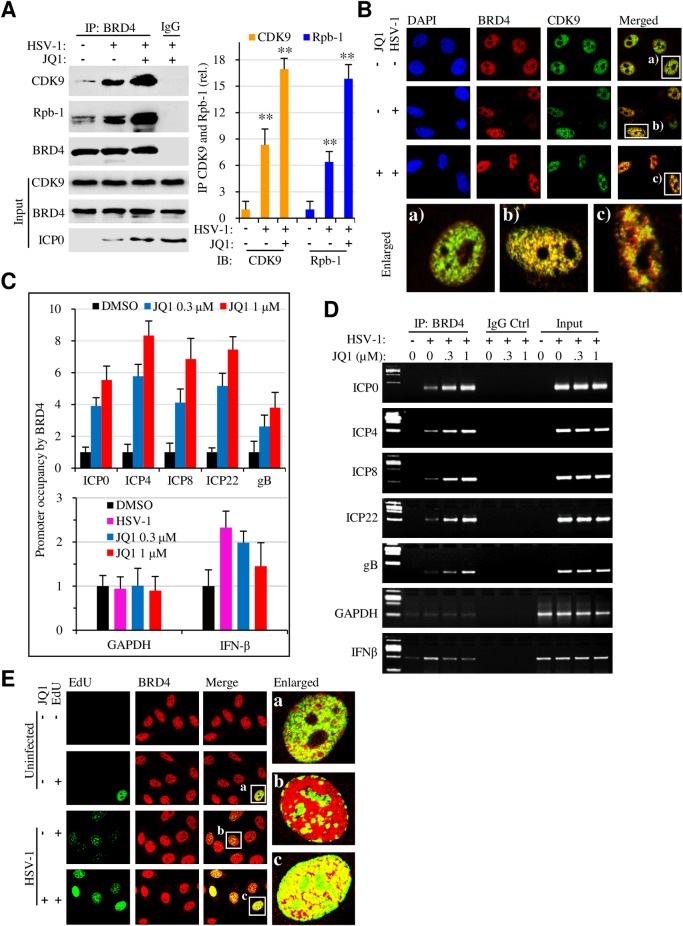
JQ1 increases BRD4 complex interaction with viral gene promoters. HeLa cell were infected with HSV-1 at 10 MOI in the absence or presence of 500 nM JQ1 for 6 hr. Protein association and their interaction with viral gene promoters were determined by immunoprecipitation, by immunostaining, and by ChIP assays, respectively. (A) JQ1 promotes protein complex formation involving BRD4, CDK9, and Rbp-1/RNAP II. Right panel bar graphs represent quantitative measurement of protein band intensities from immunoprecipitation assay. (B) JQ1 promotes BRD4 co-localization with CDK9. The enlarged areas, labeled as a, b, and c, are presented underneath the images. DAPI (blue) staining shows the nuclei. (C, D) BRD4 association with viral and host gene promoters by qPCR measurement (C) and PCR (D). (E) JQ1 promotes viral DNA synthesis and induces BRD4 redistribution. Serum-starved HeLa cells were infected with HSV-1 (10 MOI) for 4 hr in the absence or presence of 500 nM JQ1. The cells were then labeled with EdU for another 4 hr. BRD4 (Red) was visualized by staining with anti-BRD4 antibody. Green: newly synthesized DNA.

Next, we addressed whether JQ1 promoted protein association with viral gene promoters. Compared with HSV-1 infected controls, increased amount of CDK9 and Rpb-1 proteins was detected in the anti-BRD4 immunocomplexes from JQ1-treated samples (Fig 6A), an observation that was supported by immunofluorescence studies (Fig 6B). Concomitantly, there was increased recruitment of BRD4 to viral gene promoters, including ICP0, ICP4, ICP22, and gB genes in the JQ1-treated samples (Fig 6C and 6D). The results therefore indicated that JQ1 promoted protein complex formation required for HSV gene transcription.

To provide further evidence on the findings, we studied whether JQ1 effect on viral DNA synthesis by performing EdU incorporation study (Fig 6E). EdU incorporation remained scarce in serum starved cells, while HSV-1 infection promoted EdU incorporation. The staining became more intense and profound in JQ-treated samples. More importantly, BRD4 staining showed strong co-localization of viral DNA synthesis with BRD4 staining in those samples, indicating that JQ1 reallocated BRD4 and, likely, the transcriptional complex to viral gene transcription.

### BRD4 regulates HSV infection by modulation of RANP II phosphorylation through CDK9

**Fig 7:**
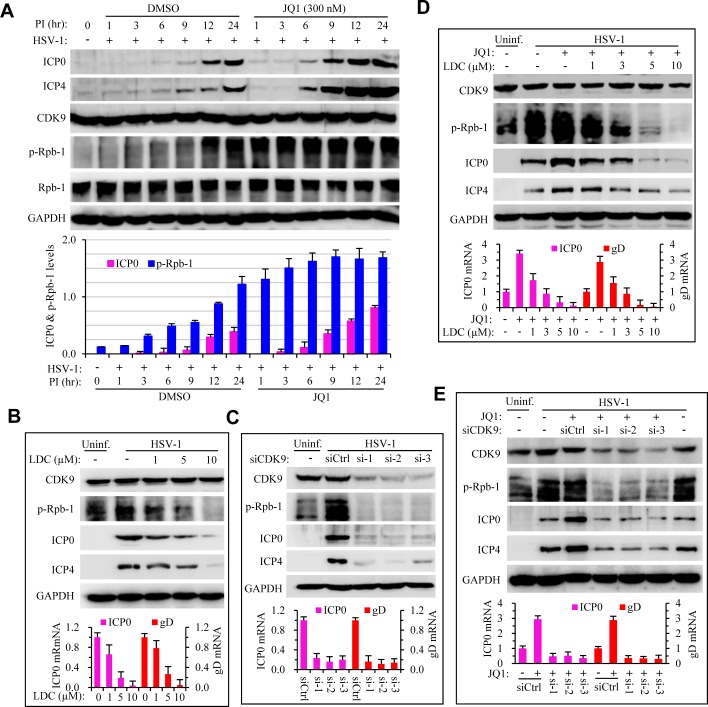
Requirement of CDK9 kinase activity for HSV-1 infection. (A) JQ1 treatment promotes HSV-1 infection and increases the phosphorylation of Rbp-1/RNAP II. HeLa cells were infected with HSV-1 (1 MOI) in the absence or presence of 300 nM JQ1 for 24 hr. Protein expression and modification was determined using specific antibodies by immunoblotting. Lower panel bar graph represents quantitative measurement of ICP0 and phosphorylated Rpb-1 band intensities relative to GAPDH. (B) CDK9 inhibitor LDC000067 (LDC) dose-dependently blocks Rbp-1 phosphorylation (upper panels) and viral gene expression, determined by western blotting and qPCR, respectively. (C) Knock down of CDK9 expression blocks Rbp-1 phosphorylation and viral gene expression, determined by western blotting and qPCR, respectively. (D) CDK9 inhibitor LDC000067 (LDC) abolishes JQ1 effect on Rbp-1 phosphorylation and HSV-1 gene expression. (E) Knock down of CDK9 expression diminishes JQ1 effect on Rbp-1 phosphorylation and viral gene expression.

BRD4 plays a diverse role in modulating viral infection by RNA and DNA viruses. Chiefly, Brd4 regulates CDK9 and RNAP II phosphorylation during viral infection [29–32]. Inhibition of CDK9 has been shown to block DNA virus infection, including HSV-1 [33,34]. The role of BRD4 in HSV infection was therefore finalized by the demonstration of CDK9 in Rpb-1 phosphorylation using a specific antibody for phospho-Ser2/Ser5 of Rpb-1 CTD. HSV infection promoted Rpb-1 phosphorylation at 3 hr PI. Treatment with JQ1 caused more intense phosphorylation of Rpb-1 which correlated with increased HSV infection in those samples (Fig 7A). Treatment with LDC000067, a CDK9 specific inhibitor, dose dependently decreased Rpb-1 phosphorylation and HSV infection (Fig 7B), which was consistent with results from RNAi studies. Moreover, we found that depletion of CDK9 expression by RNAi obliterated HSV-induced Rpb-1 phosphorylation as well as HSV infection (Fig 7C). Importantly, both treatments also annulled the enhancement effect of JQ1 on HSV infection (Fig 7D and 7E). Those results together demonstrated that BRD4 regulates HSV infection by recruiting transcriptional factors for viral gene expression. BD1 inhibitors augment HSV infection by devoting those factors more efficiently to sites of viral gene transcription.

## Discussions

**Fig 8:**
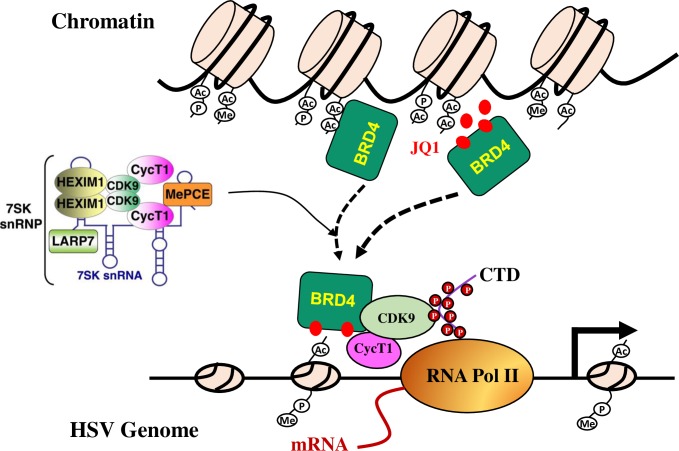
Model of BRD4 regulation of herpes simplex virus replication. BRD4 participates in epigenetics regulation by recognition of acetyl lysine residues with its bromodomains (BD1, BD2). BRD4 also regulates the transcription of cellular and viral genes by recruitment of the positive transcription elongation factor (P-TEFb) and transcriptional factors. P-TEFb is itself under a stringent control by the inhibitory 7SK small nuclear ribonucleoprotein (7SK snRNP) complex. Infection by HSV-1 or HSV-2 causes BRD4 relocation and rededicates BRD4 and the protein complex to viral gene transcription. JQ1 (red dots) or BD1 domain proteins functions by dislodging BRD4 from chromatin association.

Human herpes simplex viruses are members of Herpesviridae that have the ability to have a productive infection cycle in epithelial cells. The viruses also have the ability to establish latency where they persist in the sensory neurons. Upon infection of an epithelial cell, the viral nucleocapsid is transported along the microtubules to the nuclear pore to release the viral genome into the nucleus. The linear viral DNA circularizes rapidly in the nucleus and undergoes chromatinization and modifications characteristic of euchromatin [7,8,35]. The modifications are related to viral gene transcription because inhibition of protein methylation reduced viral gene expression [11,36], while reagents that promote histone acetylation resulted in enhanced viral replication [37]. By screening an epigenetics compound library, we discovered several bromodomain inhibitors that promote HSV infection. Those compounds modulate chromatin acetylation process since they prevent chromatin readers from binding to acetylated chromatin and interfere with histone modification. Unlike HDACi that promote HSV replication by increasing histone acetylation, JQ1 treatment did not affect the pattern of histone modifications (Fig D in S1 Text). The effect was unrelated to c-Myc suppression by c-Myc (Fig B in S1 Text). Instead, our data support a model of BRD4 regulation of HSV replication (Fig 8). HSV infection induces BRD4 association with components of transcriptional regulation machinery for viral gene transcription. BD1 domain inhibitors like JQ1 dislodge BRD4 from cellular chromatin, therefore can facilitate this association, resulting in protein complex relocation to viral gene promoters for more effective replication.

Transcription elongation has been recognized as a rate-limiting step for the expression of signal-inducible genes. By recruitment of positive transcription elongation factor P-TEFb, the bromodomain-containing protein BRD4 plays a critical role in regulating transcription elongation of a vast array of genes for multiple cellular processes and responses [38]. Here we show that bromodomain inhibition results in enhanced association of BRD4 with viral gene promoters, underscoring the importance of BRD4 in HSV infection, which was consistent with previous observations since the virus has the ability to repress host transcription by diversion of the host Pol II transcription machinery to the viral genome [39,40]. The ubiquitously expressed BRD4 is an epigenetic reader and transcriptional regulator that bookmarks active genes during mitosis and serves as a scaffold for transcription factors [41–45]. We found that BRD4 recruits transcription factors including CDK9 to viral gene promoters, which may lead to optimization of HSV-1 gene expression [33,46,47]. BRD4 was shown to function as a cellular adaptor in retaining HPV genome during mitosis [16] and is essential for viral DNA replication [15]. BRD4 blocks the recruitment of transcriptional regulators to viral gene promoters for HPV and HIV-1 latency [43,48–50]. BRD4 depletion or inhibition increases HIV-1 replication [18]. JQ1 reactivates HIV from latency by antagonizing BRD4 inhibition of Tat-transactivation [18,32,51–53]. It will be interesting to determine whether JQ1 or BRD4 plays a role in HSV reactivation. Nonetheless, this study demonstrate BRD4 is dual functional protein in epigenetic regulation and viral replication.

It is known that hexamethylene bisacetamide (HMBA), a potent inducer of differentiation of tumor cells, is capable to enhance HSV replication [54–56]. The mechanism has yet to be defined. One peculiar feature of its purported mode of action on virus gene expression kinetics is that the effect is transient and requires a short exposure (1.5 to 5 h) to the agent early after infection using a VP16-null mutant [55,57]. Various signaling events, UV light, or chemicals such as HMBA cause transiently release of P-TEFb from its inhibitory apparatus, resulting in host gene transcription [58,59]. P-TEFb is sequestered by small nuclear ribonucleoprotein (7SK snRNP) and remains inactive in quiescent cells [60,61]. Free P-TEFb, which is required for acute response and cell growth, can be recruited to RNAP II via the super elongation complex (SEC), BRD4, or transcription factors. We show that JQ1 treatment enhances complex formation involving BRD4-P-TEFb-RNAP II. It is likely that HMBA and JQ1 share a common mechanism in promoting HSV replication.

The compounds that promote HSV infection were discovered by screening an epigenetics compound library. An obvious advantage of screening a compound library with defined targets is that it allows rapid but definitive dissection of biological events like HSV infection with ease since the compounds, structurally diverse, serve as probes to cross-validate each other. The identification of BRD4 as an essential factor in HSV lytic infection uncovers a novel target for antiviral drug research. BET inhibitors are currently developed for tumor therapy [62,63]. Our results raise questions that those agents may exacerbate existing herpes infection. With recent approval of an oncolytic herpes virus for the treatment of certain tumors, JQ1 and similar compounds nonetheless may have potential application in tumor therapy with oncolytic HSV vectors.

## Materials and Methods

### Cells and viruses

Vero cell line (African green monkey kidney epithelial cells, ATCC CCL-81), HeLa cell line (ATCC, CCL-2), HEp-2 cell line (ATCC, CCL-23) and human neuroblastoma SK-N-SH cells (ATCC, HTB-11) were purchased from ATCC (Manassas, VA) or from Cell Bank of Chinese Academy of Sciences (Shanghai, China). Mouse embryo fibroblast (MEF) cells (isolated from C57BL/6J mouse embryos) were prepared in the lab following published protocol [64]. The cells were cultured in DMEM (high glucose) supplemented with 10% heat-inactivated fetal bovine serum (FBS), sodium pyruvate and non-essential amino acids (Life Technologies, Carlsbad, CA) in a humidified incubator at 37°C with 5% CO_2_. HSV-1 strain F and HSV-2 strain G were propagated and titrated on Vero cells.

### Reagents and Antibodies

An epigenetics compound library (Catalog No. L1900) was purchased from Selleck Chemicals China (Shanghai). This library contains cell permeable inhibitors of epigenetic enzymes including histone deacetylases (HDACi), lysine demethylases, histone acetyltransferases (HATs), DNA methyltransferases (Dnmts), and the epigenetic reader domain inhibitors. Trichostatin A (TSA), PFI-1, JQ1, TG101348, RVX-208, and LDC000067 (CDK9 inhibitor) were also purchased from Selleck Chemicals. (-)-JQ1 was purchased from MedChem Express. Antibodies to BRD4 (Abcam, ab128874), CDK9 (Santa Cruz, sc-13130), Rpb-1 CTD (Cell Signaling Technology, 2629), phospho-Rpb1 CTD (Cell Signaling Technology, 13546, Ser2/Ser5 specific), c-Myc (Bioworld Technology, BS2462), histone H3 (Beyotime Institute of Biotechnology, AH433), phospho-histone H3 (Cell Signaling Technology, 3377, ser10), acetylated-histone H3 (Millipore, DAM1776434, Lys9/Lys14), GAPDH (Bioworld Technology, MB001) were obtained commercially. Antiserum to ICP4 (HSV-1 and -2; GenScript, Nanjing), and to ICP0 (HSV-1 and -2; ABmart, Shanghai) were prepared in New Zealand rabbits using commercial sources. Horse radish peroxidase (HRP)-conjugated secondary antibodies were purchased from Bio-Rad. Alexa Fluor 488 (Green) conjugated anti-mouse IgG and Alexa Fluor 568 (Red) conjugated anti-rabbit IgG were purchased from Life Technologies. Protein G agarose beads were purchased from Roche Diagnostics.

### Compounds screen and virus infection assays

We first determined cytotoxic effect of the compounds on Vero cells using an MTT assay as we previously described [65]. To screen for their activity on HSV infection, a DMSO stock of a tested compound was freshly diluted into culture medium and tested in triplicate at the maximal non-toxic concentration at 2 hr prior to inoculation. HSV-2 at 1 MOI was used for the screening. The compounds or DMSO at 0.1–0.2% were left in the medium throughout the infection process. We measured cell viability at 48–60 hr PI using the MTT assay to quickly measure the cytopathic effect by HSV infection. A plaque forming assay (PFU) was then performed if a compound showed selective effect on HSV infection.

### DNA and transfection

pCMV2-FLAG-BRD4 was purchased from Addgene (#22304). The plasmid encodes full length BRD4 (NM_058243) for mammalian expression [49]. The DNA corresponding to BD1 (aa 1–196), BD1/2 (aa 1–640), and BD1 deletion (ΔBD1, aa 197–1362) was inserted into p3xFLAG-CMV-24 (Sigma) using standard protocols of molecular biology. For transfection studies, the DNA were transfected using Lipifectamine-2000 (Life Technologies). The cells were used at 24 hr post transfection for different assays.

### RNA interfering

The oligos of small interfering RNA (siRNA) targeted human BRD4 or CDK9 were synthesized by GenePharma (Shanghai, China). For gene knockdown experiments, cells were plated in 6- or 24-well plates (Corning) 24 hr before transfection. Cells were transfected using Lipofectamine-2000. The cells were used at 48–72 hr after transfection for further experiments.

BRD4-siRNA #1: 5’-CUCCCUGAUUACUAUAAGATT-3’ and

                            5’-UCUUAUAGUAAUCAGGGAGTT-3’

BRD4-siRNA #2: 5’-GGAGAUGACAUAGUCUUAATT-3’ and

                            5’-UUAAGACUAUGUCAUCUCCTT-3’

BRD4-siRNA #3: 5’-GCACAAUCAAGUCUAAACUTT-3’ and

                            5’-AGUUUAGACUUGAUUGUGCTT-3’

BRD2-siRNA #1: 5’-CACGAAAGCUACAGGAUGU-3’ and

                            5'-ACAUCCUGUAGCUUUCGUG-3’

BRD2-siRNA #2: 5’-GGGCCGAGUUGUGCAUAUA-3’

                            5'-UAUAUGCACAACUCGGCCC-3’

BRD3-siRNA #1: 5’-AAUUGAACCUGCCGGAUUA-3’ and

                            5'-UAAUCCGGCAGGUUCAAUU-3’

BRD3-siRNA #2: 5’- CGGCUGAUGUUCUCGAAUU-3’ and

                            5'-AAUUCGAGAACAUCAGCCG-3’

c-Myc-siRNA #1: 5'-ACGGAACUCUUGUGCGUAA-3’ and

                            5'-TTACGCACAAGAGUUCCGU-3’

c-Myc-siRNA #2: 5’-GAACACACAACGUCUUGGA-3’ and

                            5'-UCCAAGACGUUGUGUGUUC-3’

CDK9-siRNA #1: 5’-GGAGAAUUUUACUGUGUUUdTdT-3’ and

                            5’-AAACACAGUAAAAUUCUCCdTdT-3’

CDK9-siRNA #2: 5’-CCGCUGCAAGGGUAGUAUAdTdT-3’ and

                            5’-UAUACUACCCUUGCAGCGGdTdT-3’

CDK9-siRNA #3: 5’-UAGGGACAUGAAGGCUGCUAAdTdT-3’ and

                            5’-UUAGCAGCCUUCAUGUCCCUAdTdT-3’

### Immunoprecipitation and western blot analysis

Cell lysates were collected by centrifugation after cell lysis using a buffer containing 50 mM Tris-HCl (pH 7.4), 150 mM NaCl, 1% NP-40, and a cocktail of protease inhibitors (Roche). For immunoprecipitation studies, cell lysates were incubated at 4°C for 2 hr with a capture antibody or a control antibody, followed by overnight incubation with protein G-agarose beads. The immunocomplexes were collected by centrifugation, then washed with ice-cold PBST (PBS-0.02% Tween-20), and separated by SDS-PAGE.

### Chromatin immunoprecipitation (ChIP) assay

A modified ChIP assay was performed to determine protein and viral DNA interactions [66]. Briefly, HSV-1-infected cells (2x10^7^) were washed with ice-cold PBS for three times and consequently cross-linked using 1% formaldehyde. The reaction was quenched by adding glycine (125 mM). After rinse with ice-cold PBS, the cells were collected into a cell lysis buffer containing 5 mM PIPES (pH 8.0), 1% SDS, 1 mM EDTA, and protease inhibitors. The mixture was incubated on ice for 15 min followed by sonication at an amplitude of 30 on a 15-sec on and 10-sec off cycle for 20 min to shear the DNA. The supernatants were collected by centrifugation and were used immediately or stored at -80°C. For immunoprecipitation, the supernatants were diluted using a ChIP dilution buffer (Beyotime) and precleared using salmon sperm DNA/protein G agarose beads (Roche). The protein-DNA complexes were precipitated with an indicated antibody and protein G beads at 4°C for overnight. The immune complexes were washed with a low salt buffer containing 150 mM NaCl in buffer A (2 mM EDTA, 1% Triton X-100, 0.1% SDS, 20 mM Tris-HCl, pH 8.1), followed by a high salt buffer (500 mM NaCl in buffer A), and then LiCl wash buffer (250 mM LiCl, 1 mM EDTA, 1% NP-40, 1% deoxycholate, 10 mM Tris-HCl, pH 8.1). The complexes were eluted with an elution buffer (1% SDS, 0.1 M NaHCO_3_). Contaminating RNA was removed by treating with 10 μg/ml RNase A. The complexes were then incubated at 65°C for overnight to reverse the cross-linking and then with proteinase K at 55°C for 1 hr to remove proteins. The DNA was purified by phenol chloroform extraction, followed by ethanol precipitation, and used for analysis of DNA-protein association using real-time and PCR. The primers are listed in S1 Table.

### RT-PCR and real-time PCR assay

For RT-PCR studies, total RNA was extracted using TRIzol reagent (Life Technologies). One microgram RNA was reverse transcribed into cDNA using AMV reverse transcriptase. Real-time PCR was performed using SYBR Green PCR Master Mix (Q141-02/03, Vazyme, Nanjing, China) on an ABI 7300 real-time PCR system (Applied Biosystems) and data was analyzed using the 2^-ΔΔCt^ method to obtain relative abundance [67]. The GAPDH Ct level was used as an internal control for value normalization.

### Immunofluorescence and confocal microscopy assays

Cells cultured on coverslips were fixed with 4% paraformaldehyde for 10 min at room temperature (RT). The cells were permeabilized at RT by treating with 0.2% Triton X-100 for 10 min. The cells were blocked with normal goat serum for 30 min and then stained by incubation with antibody to BRD4 (1:500 dilution), to Rpb-1 (1:700 dilution), or to CDK9 (1:500 dilution) at 4°C for overnight. Alexa Fluor conjugated secondary antibodies (1:1000) were used for visualization. Cell nuclei were stained with 1 μg/ml DAPI for 10 min. The images were collected on an Olympus FluoView FV10i confocal microscope.

### HSV genome labeling and Click chemistry study

We used EdU incorporation method to measure viral genome replication [28]. Briefly, HeLa cells grown on glass coverslips were serum-starved in DMEM containing 0.5% FBS for 24 hr to arrest cells at G_0_ stage. The cells were then infected with HSV-1 at 10 PFU/cell for 4 hr, at which time the medium was replaced with fresh DMEM containing 2% FBS and 5 μM EdU and cultured for another 4 hr. EdU-labeled DNA was conjugated by reaction with Alexa Fluor 488 azide using the Click-iT EdU imaging kit (Life Technologies). The nuclei were stained with DAPI. BRD4 redistribution was detected with confocal microscope.

### Statistical analysis

Statistical analysis was performed using SPSS 17.0 software package. Data were analyzed by paired T test. P values equal to or less than 0.05 were considered statistically significant.

## Supporting Information

S1 Text **Fig A. Bromodomain inhibitors on HSV-1 and HSV-2 infection on different cells** Cells in 6-well plates were treated with a test compound of with 0.1% DMSO (Mock, solvent control) for 2 hr. The concentrations used are as the following: JQ1, 300 nM; PFI-1, 500 nM; TSA, 150 nM; (-)-JQ1 at 300 nM. The cells were then infected with HSV-1 or with HSV-2 at 1 MOI for 24 hr. Virus production were determined by plaque assay on Vero cells. (A) HeLa cells. (B. HEp-2 cells. (C) SK-N-SH cells. (D) Mouse embryonic fibroblast cells (MEF). **Fig B. Suppression of c-Myc expression has no effect on HSV infection.** (A) HeLa cells were treated with siRNA targeting BRD4 or c-Myc for 48 hr, or with JQ1 for 24 hr. Levels of c-Myc were determined by qPCR using GAPDH as a control. (B, C) Suppression of c-Myc directly by siRNA or indirectly by BRD4 suppression or JQ1 (300 nM) treatment, did not affect HSV-1 infection or JQ1 effect on HSV-1 and HSV-2 infection. For infection assay, the cells were treated with siRNA for 48 hr prior to infection. si-cMyc/JQ1: JQ1 at 300 nM was added during virus infection of si-cMyc treated cells. **Fig C. Selectivity of oligos used for ChIP assay.** A. Schematic drawing of primer paired used for ChIP assay and controls. Transcription start site (TSS) is marked using a red arrow. Oligos targeting the promoter regions (PR), upstream non-promoter region (UNPR), and downstream non-promoter region (DNPR) for ICP0, ICP4, and gB were used for the study. Oligo sequences are listed in S1 Table. B. Agarose gel electrophoresis of DNA fragments after sonication for 10 min (lane 1) and 20 min (lane 2), respectively. Sample of lane 2 was used for the study. C. The sample was immuneprecipitated with anti-BRD4 antibody. BRD4-associated viral DNA were amplified using oligos targeting the promoter region (PR), upstream non-promoter region (UNPR), or downstream non-promoter region (DNPR). **Fig D. JQ1 does not cause histone modifications.** HeLa cells were solvent-treated (mock, 0.1% DMSO), treated with JQ1 at 300 nM, infected with HSV-1 (1 MOI) in the absence (HSV-1) or presence of 300 nM JQ1 (H+J). As a positive, TSA at 150 nM was included. The samples were treated or infected for 24 hr. Histone-3 acetylation (Ac-H3), ser-10 phosphorylation (p-H3), as well as viral ICP0 and ICP4 protein expression was detected by immunoblotting. GAPDH was used as a loading control.
(TIF)

S1 Table List of primer pairs used for gene detection (DOCX)
