# Supplementary figures and images for: An Epigenetic Compound Library Screen Identifies BET Inhibitors That Promote HSV-1 and -2 Replication by Bridging P-TEFb to Viral Gene Promoters through BRD4
Source: PLoS Pathog. 2016 Oct 20;12(10):e1005950. doi: 10.1371/journal.ppat.1005950 (PMC5072739; doi:10.1371/journal.ppat.1005950)

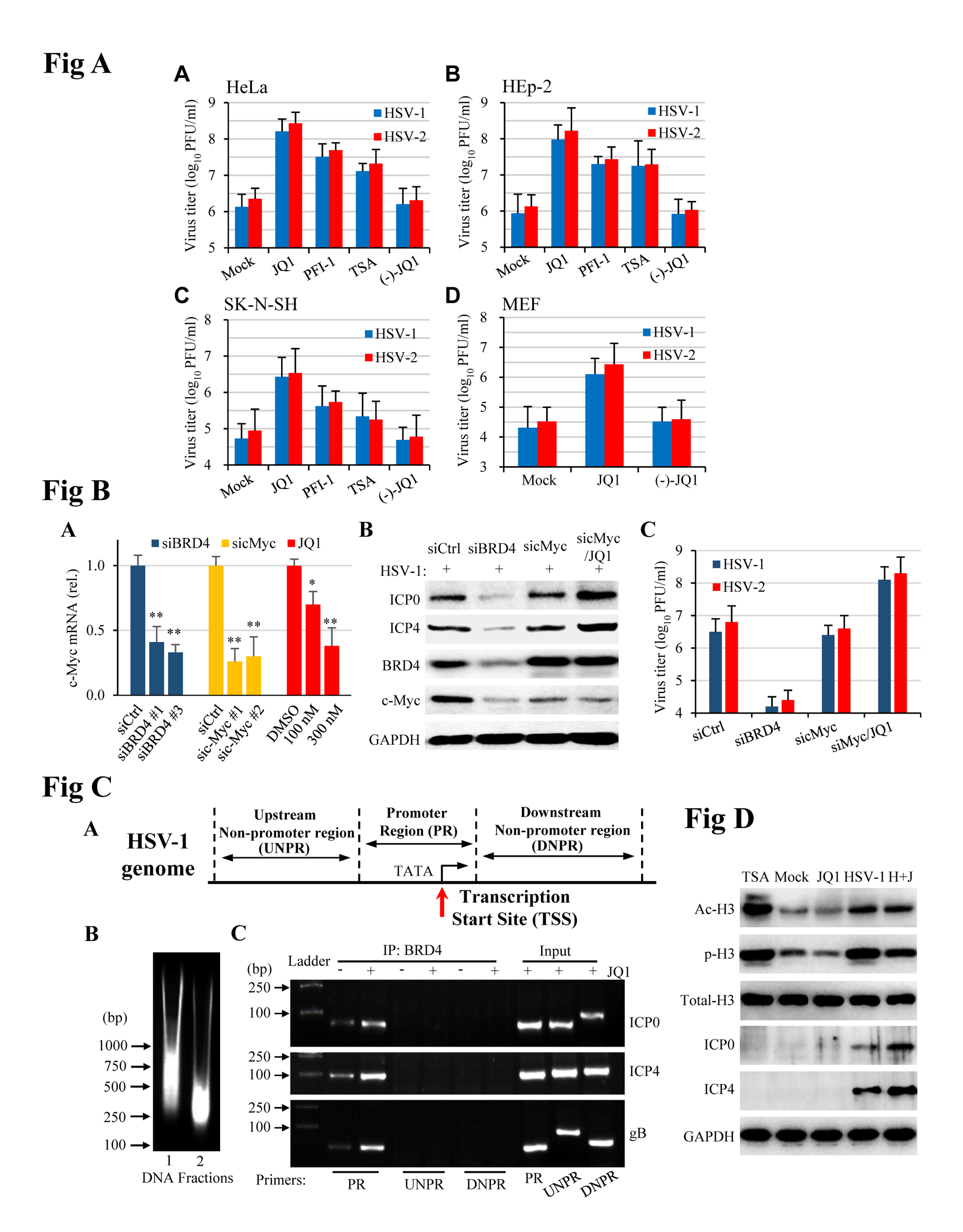

Supplement: S1 Text — Fig A. Bromodomain inhibitors on HSV-1 and HSV-2 infection on different cells Cells in 6-well plates were treated with a test compound of with 0.1% DMSO (Mock, solvent control) for 2 hr. The concentrations used are as the following: JQ1, 300 nM; PFI-1, 500 nM; TSA, 150 nM; (-)-JQ1 at 300 nM. The cells were then infected with HSV-1 or with HSV-2 at 1 MOI for 24 hr. Virus production were determined by plaque assay on Vero cells. (A) HeLa cells. (B. HEp-2 cells. (C) SK-N-SH cells. (D) Mouse embryonic fibroblast cells (MEF). Fig B. Suppression of c-Myc expression has no effect on HSV infection. (A) HeLa cells were treated with siRNA targeting BRD4 or c-Myc for 48 hr, or with JQ1 for 24 hr. Levels of c-Myc were determined by qPCR using GAPDH as a control. (B, C) Suppression of c-Myc directly by siRNA or indirectly by BRD4 suppression or JQ1 (300 nM) treatment, did not affect HSV-1 infection or JQ1 effect on HSV-1 and HSV-2 infection. For infection assay, the cells were treated with siRNA for 48 hr prior to infection. si-cMyc/JQ1: JQ1 at 300 nM was added during virus infection of si-cMyc treated cells. Fig C. Selectivity of oligos used for ChIP assay. A. Schematic drawing of primer paired used for ChIP assay and controls. Transcription start site (TSS) is marked using a red arrow. Oligos targeting the promoter regions (PR), upstream non-promoter region (UNPR), and downstream non-promoter region (DNPR) for ICP0, ICP4, and gB were used for the study. Oligo sequences are listed in S1 Table. B. Agarose gel electrophoresis of DNA fragments after sonication for 10 min (lane 1) and 20 min (lane 2), respectively. Sample of lane 2 was used for the study. C. The sample was immuneprecipitated with anti-BRD4 antibody. BRD4-associated viral DNA were amplified using oligos targeting the promoter region (PR), upstream non-promoter region (UNPR), or downstream non-promoter region (DNPR). Fig D. JQ1 does not cause histone modifications. HeLa cells were solvent-treated (mock, 0.1% DMSO), t [file ppat.1005950.s001.tif]
