# Supplementary material for: An Epigenetic Compound Library Screen Identifies BET Inhibitors That Promote HSV-1 and -2 Replication by Bridging P-TEFb to Viral Gene Promoters through BRD4
Source: PLoS Pathog. 2016 Oct 20;12(10):e1005950. doi: 10.1371/journal.ppat.1005950 (PMC5072739; doi:10.1371/journal.ppat.1005950)
Supplement: S1 Table — (DOCX) [file ppat.1005950.s002.docx]

**S1 Table A. Primer sequences for ChIP and real-time PCR studies ***

| **Gene** | **Primers for ChIP analysis** | **Location** | **Production size** |
| --- | --- | --- | --- |
| ICP0^PR^ | 5’-ATAAGTTAGCCCTGGCCCCGA (F)  5’-GCTGCGTCTCGCTCCG (R) | -29 to +35 bp | 65 bp |
| ICP0^UNPR^ | 5’-TCTAACGTTACACCCGAGGC (F)  5’-AGAGCGGCTTGGTGCG (R) | -900 bp | 69 bp |
| ICP0^DNPR^ | 5’- CGTGTGCACGGATGAGATCG (F)  5’- GCGCAATTGCATCCAGGTT (R) | +1100 bp | 100 bp |
| ICP4^PR^ | 5’-CTATATGAGCCCGAGGACGC (F)  5’-CGTCTGACGGTCTGTCTCTG (R) | -31 to +68 bp | 100 bp |
| ICP4^UNPR^ | 5’- CGCGTACGAACACGTCGAT (F)  5’- TTTATCACCACCATGGCCC (R) | -2000 bp | 110 bp |
| ICP4^DNPR^ | 5’- GGAGTTTCTGGGGCTGCTC (F)  5’- GTCGCACGCCAGGTAGG (R) | +1300 bp | 127 bp |
| gB^PR^ | 5’-TGGGTGGAGTGATCAAAGAG (F)  5’- GCATCACCCATCGCTTCT (R) | -257 to -201 bp | 57 bp |
| gB^UNPR^ | 5’-TCGTCAGGTGGTGGTCGTA (F)  5’- TGGACGCCCTAATAATCGGC (R) | -900 bp | 90 bp |
| gB^DNPR^ | 5’-CAGTCGCCAGCACAAACTC (F)  5’-ACGACGGTAAACTGCATCGT (R) | +1000 bp | 76 bp |
| GAPDH^PR^ | 5’- TTCGACAGTCAGCCGCATCTTCTT (F)  5’- CAGGCGCCCAATACGACCAAATC (R) | +44 to +153 bp | 110 bp |
| IFN-β^PR^ | 5’- TAGTCATTCACTGAAACTTTA (F)  5’- AGGTTGCAGTTAGAATGTC (R) | -103 to +96 bp | 200 bp |
| ICP8^PR^ | 5’-CCACGCCCACCGGCTGATGAC (F)  5’-TGCTTACGGTCAGGTGCTCCG (R) | -107 to +20 bp | 128 bp |
| ICP22^PR^ | 5’-TATTAGGGCGAAGTGCGAGC (F)  5’-CTTTATGTGCGCCGGAGAGA (R) | -28 to +72 bp | 101 bp |

| **Gene** | **Primers for real-time PCR** | **Accession #** | **Product size** |
| --- | --- | --- | --- |
| ICP0 | 5’-CGTGTGCACGGATGAGATCG  5’-GCGCAATTGCATCCAGGTT | GU734771 | 100 bp |
| gD | 5’-TTACTACGCCGTGTTGGAGC  5’-TGTAGGGTTGTTTCCGGACG | GU734771 | 110 bp |
| BRD2 | 5’-CGGAGGTGTCCAATCCCAAA  5’-TGTGATAATCCGGTAGACCCAG | NM_005104 | 153 bp |
| BRD3 | 5’-CCACCCCTGTACCAACCATC  5’-GGGAGTGGTTGTGTCTGCTT | NM_007371 | 161 bp |
| BRD4 | 5’-GCGTTTCCACGGTACCAAAC  5’-ATGACAGGGGTCTGGACGAT | NM_058243 | 142 bp |
| c-Myc | 5’-GCTCGTCTCAGAGAAGCTGG  5’-GCTCAGATCCTGCAGGTACAA | NM_002467 | 117 bp |
| GAPDH | 5’-ACAGTCAGCCGCATCTTCTT  5’-ACGACCAAATCCGTTGACTC | NM_002046 | 97 bp |

* The oligo sequences were based on HSV-1 strain F (GenBank accession number GU734771).

F: forward; R: reverse. PR: promoter region; UNPR: upstream non-promoter region; DNPR: downstream non-promoter region; Location: refers to region relative to transcription start site.
